# Video Game Streaming in Young People and Teenagers: Uptake, User Groups, Dangers, and Opportunities

**DOI:** 10.3390/healthcare9020192

**Published:** 2021-02-10

**Authors:** Luis Javier Cabeza-Ramírez, Guzmán Antonio Muñoz-Fernández, Luna Santos-Roldán

**Affiliations:** Department of Statistics, Econometrics, Operations Research, Business Organization and Applied Economics, Faculty of Law, Business and Economic Sciences, University of Córdoba, Puerta Nueva s/n, 14071 Córdoba, Spain; guzman.munoz@uco.es (G.A.M.-F.); td1sarol@uco.es (L.S.-R.)

**Keywords:** digital motivation, cyber behaviour, live streaming, cluster analysis, video games, e-health, problematic online gaming and gambling

## Abstract

In recent years, live video streaming platforms for video games have been gaining popularity. These types of services, which enable anyone to broadcast and consume live content, are revolutionising the current video game landscape. Users approach the emergence of and participation in these platforms driven by a range of motivations. It is essential to characterise the different forms of participation in services such as Twitch to evaluate the phenomenon and reflect on its advantages and disadvantages. To that end, a survey was carried out of 580 young people and adolescents aged between 14 and 24. The aim of this study is thus to explore the uptake of these platforms, as well as identify user groups, distinguish between different motivations, and address the associated benefits and harms. Applying a methodology based on factor analysis and cluster analysis, user profiles were characterised according to their specific features, gaming/viewing hours, self-perception of their skill level as a player, devices used, and type or genre of video game. Four subgroups of gamers/viewers were thus identified: casual, social, hobby, and problematic. The results showed that older users and female users feature more prominently in the first two groups, as do those spending less time on video games and live streaming platforms. Conversely, in the hobby and problematic groups, we observe just the opposite. The existence of profiles at possible risk of addiction underlines how, at a preventive level, there is a need for more in-depth research on these types of services and greater public awareness of the dangers of uncontrolled use.

## 1. Introduction and State of the Research

Over the course of human history, if there is one society that has championed the prioritisation of free time as a need (Art. 24 [1]), it is probably that of the current era. Leisure time fosters personal development and can take different forms. Of these, video games have become one of the most commercial and most compelling types of entertainment [2].

Playing video games is a common activity among young people and adolescents [3]. There is an ongoing debate as to people’s motivations for gaming [4], as well as the potential benefits [5,6] and disadvantages [7]. The earliest studies on motivation associate this hobby with simple fun: a way of beating boredom, facing new challenges, or sharing interests [4,8]. The main benefits concern the development of social, educational, and spatial skills [9,10,11]. However, looking beyond their healthy or adaptive use, video games can also give rise to abusive use or lack of control, which could affect the daily lives of millions of users if this activity becomes predominant. Adverse effects include addictive behaviours, antisocial behaviours, reduced sleep, continual tiredness, a decline in academic or work performance, and even physical problems [12,13].

The world of video games is currently in the midst of a genuine revolution, ushered in by two relatively recent and interconnected phenomena: the rise of e-sports and the development of activities related to the live streaming of video games. Crowdsourced live streaming services allow anyone to broadcast and consume live content over the internet [14]. Notable in the field of video games are platforms such as Twitch, which surpassed 11.9 billion hours streamed in 2018, with an average of 1.25 million concurrent viewers in the first quarter of 2019 alone [15]. Moreover, as a result of the containment measures against COVID-19, the number of viewers and of professionals or amateurs currently streaming content continues to rise [16]. The big companies are battling it out over this new form of entertainment. Alongside Twitch, which is owned by Amazon, there are other platforms such as YouTube Gaming (Google) (Google LLC., Mountain View, CA, USA), Mixer (Microsoft) (Microsoft Corporation Albuquerque, NM, USA), Facebook Gaming (Facebook) (Facebook, Inc., Menlo Park, CA, USA), and Caffeine (ex-executives of Apple TV). On such platforms, users are not limited to being mere spectators, they interact with other participants, comment on games or strategies, make donations, and subscribe. They may also take on the role of content developers or streamers, in order to stream events, test games, disseminate and comment on games, as well as other activities directly or indirectly related to video games [17].

The positive and negative elements associated with video games also apply to live streaming services [18]. The factors linked to the risk or opportunity arising from the consumption of live video game streaming may be heterogeneous among individuals [19]. Previous research has primarily focused on the positive or negative aspects associated with the rise of this new phenomenon [18,20,21,22], but no studies to date have identified user groups or differences between them and their perceptions. As Jeong, et al. [19] point out, the identification of homogeneous subgroups can contribute to a better understanding of gaming and viewing behaviour. In this regard, identifying subgroups can help shed light on the heterogeneity of the population under study. This type of analysis has been undertaken in many different contexts and has been used in a range of research areas, reflecting its effectiveness for exploratory data analysis [23]. It has proven especially useful in research related to the identification of potential risk groups; for example, it has been applied to determine the profiles of people who engage in online betting [24], to explore behaviours related to the binge watching of television series [25], or more recently, to assess the psychological impact of the COVID-19 pandemic [26].

In recent years, some promising lines have emerged within the research on live streaming that point to the professional opportunities linked to streaming [21,27,28,29,30], or the potential benefits in relation to learning [31,32]. Nevertheless, as Woodcock and Johnson [33] warn, making the transition from a hobby to a way of earning a living requires marathon gaming sessions or for streamers to produce content even when they are tired or not in the mood to do so. Paradoxically, some of the most pernicious effects of video games are associated precisely with time and usage patterns [6,34,35], neuropsychiatric deficits, and certain types of video games [36]. Therefore, the present study aims to explore the profiles of users (under the age of 25) of video game streaming services. The analysis seeks to identify their motivations and establish subgroups according to their characteristics relating to time spent gaming and viewing, self-perception of their skill level as a player, devices used, and perception of two additional aspects—whether they believe their hobby can be turned into a profession and whether they perceive that they derive benefits in terms of learning on live streaming platforms.

### 1.1. Video Games and Live Streaming: The Rise of the Streaming Phenomenon

The success of video games, due in part to their interactive nature [37], extends to other, related activities, with a growth in the number of users who not only spend their time gaming, but also use streaming platforms to learn, interact with other fans, and watch streams of their favourite pastime. In the third quarter of 2019, Twitch, YouTube Gaming, and Mixer combined generated 3.32 billion hours watched, more than 131 million live hours, with an average of nearly 1.6 million concurrent viewers [38]. As Lin, Bowman, Lin, and Chen [37] point out, there are three essential components to live game streaming: the public playing of the video game before a potential audience, the varying levels of interaction between streamers and viewers, and the fact that such interactions can coexist with gaming outside of streaming. Hence, some authors hold that video game streaming offers elements of utility, such as learning strategies to apply when playing, and social aspects, linked to the interaction between users [39].

The growing importance of this phenomenon is in little doubt. In this respect, Johnson and Woodcock [22] underline its impact on the entire video game ecosystem and industry, explaining how it helps to create links between developers/programmers and streamers/influencers. They claim it even changes players’ expectations regarding new product launches, influences video game design, and boosts the lifespan and visibility of older games or independent titles. Another decisive factor relates to the transition from traditional broadcast media, such as television, to new, more flexible and interactive formulas, where spectating and participation bring new life to the concepts established in studies of audiences [40]. Furthermore, the phenomenon of live streaming places a priority on the social component. Hamilton, Garretson, and Kerne [39] point out that these types of platforms are meeting places for communities of players, where the emphasis is on development and a sense of belonging, leading to the creation of intimate groupings with a small number of spectators, or large groups, usually for the streaming of major tournaments and events; in other words, we see the emergence of informal communities that socialise and participate. Along the same lines, Churchill and Xu [41] identify different subcultures dedicated to specific games.

### 1.2. Motivation in Video Games and Crowdsourced Platforms

Knowing the different motivations of different users is essential in order to gain an understanding of their behaviour and to identify distinct profiles. Motivation is one of the aspects that most strongly influences the desire to continue playing [42]. Some of the motivational factors that play a role in the process of choosing a video game to play or watch are the need for information about the game, its utility as a source of escapism and fun, as well as those relating to social integration and the strengthening of friendship networks [43,44]. However, the type of player or even their skill level is likely to influence that motivation [45]. In this regard, Bartle [46] established a now-classic taxonomy, distinguishing between achievers, or players focused on completing challenges, socialisers, oriented towards social skills, creating contacts, and social interaction, explorers, interested in understanding the details of the world set out by the video game, and killers, for whom the competitive factor predominates.

Indeed, one of the most promising avenues of research into live streaming of video games focuses on the analysis of motivation [43,44,47]; although watching others play is not exactly the same as playing, they are overlapping phenomena, since both entail consuming related content [48]. In this respect, one of the most well-established theories is the uses and gratification theory (UGT), commonly adopted in the framework of analysis of media consumption. Thus, Sjoblom and Hamari [43] distinguish between five types of motivation: cognitive (acquiring information, knowledge, comprehension), affective (seeking an emotional, pleasant, or aesthetic experience), personal integrative (enhancing credibility, confidence, and status), social integrative (enhancing connections with family, friends, and so forth), and tension-release (escape and diversion). They establish that the search for information is positively associated with the hours that the users dedicate to these types of services. In addition, they find that tension-release, social integrative, and affective motivations are also related to the time spent, although social integrative is considered the main predictor of subscription to these types of services. Using the same theoretical framework, Hilvert-Bruce et al. [49] identify additional motivations that help explain the commitment to live streaming, such as entertainment and lack of external support in real life. Within the same framework and applied to e-sports streaming, Hamari and Sjoblom [48] find a positive association between the frequency of watching and factors such as escapism, the skills of the player being watched, the novelty of the video game, and aggressive e-sports behaviour. Beyond UGT, Gandolfi [47] adds other motivations such as the attractiveness or popularity of specific streamers, or the interest in particular games or genres.

### 1.3. Positive Effects of Video Games: Opportunities Associated with Live Streaming

The literature on the utility and benefits of video games reveals enormous potential. Results have been found indicating a connection with enhanced cognitive abilities, improved coordination of senses such as sight or touch, spatial reasoning, capacity for attention and creativity [50,51], and the development of social, educational, and spatial skills [9,10,11]. Gamification is also being explored as a means of instrumentalising learning [31]: interactivity and viewer participation in video games facilitate the acquisition of knowledge in a natural way [52].

This set of benefits extends to live video platforms; however, an approach to such services requires a more in-depth exploration of what they offer. Gros, Wanner, Hackenholt, Zawadzki, and Knautz [44] point to the social component of the service, holding it to be a new type of social network of players. On the other hand, Spilker, Ask, and Hansen [40] emphasise its media aspect. As such, this dual nature should be taken into account. These days, platforms such as Twitch bring together a growing number of channels grouped into categories that mainly relate to video games, but also include sections such as IRL (in real life), dedicated to widely varying themes: chatting, special events, talk shows and podcasts, music and performing arts, to name just a few that have the biggest audience and most followers outside of video games.

In line with this dual utility, the most recent studies on video games and streaming platforms focus on the professional possibilities, where gaming stops being a hobby and becomes an occupation [21,33], or on their contribution from an educational viewpoint. Payne, Keith, Schuetzler, and Giboney [32] show how Twitch represents a unique learning paradigm and provides opportunities for instructors (streamers) to educate mass audiences in real-time, allowing teacher–student and student–student interaction, while Liao, Chen, and Shih [31] demonstrate how the instructional use of video and collaboration influence the achievement of learning based on digital games.

### 1.4. Problems and Dangers Related to Video Games and Live Streaming

Some adverse effects are related to antinormative behaviour, harassment, or dissemination of offensive or inappropriate content [53,54,55].

Misgivings about problematic gaming, especially in children, adolescents, and young people, has become a focus of growing concern among parents and caregivers [56]. As Buiza-Aguado et al. [57] point out, there is no consensus as to the clinical definition of video game addiction, although instruments have been developed to detect harmful use, culminating in the World Health Organisation’s recent inclusion of Gaming Disorder [58] as a behavioural addiction [59]. As Buiza-Aguado [56] shows, poor psychosocial functioning seems to be a key factor in the development of addictive patterns of use, patterns principally associated with males. Other factors that play a role include online gambling, time spent playing, and genre of game, as well as social and family factors.

In relation to the internet, as Fineberg et al. [60] point out, while its positive and adaptive uses are highly valued, a spectrum of uncontrolled use has also been acknowledged, including disordered online behaviour (excessive use of online video games, uncontrolled viewing of pornography, compulsive buying, or addiction to social networks). These are all factors that may be associated with functional impairment, loss of productivity or poor academic performance, and even mood disorders and anxiety [58,61]. Although there is a growing number of analyses of their impact on young people and adolescents [62,63], the excessive use of live streaming services has received little attention to date [18,64].

## 2. Materials and Methods

The methodology used is based on primary data collected through an online survey administered via the Surveymonkey platform.

### 2.1. Study Design and Process

The study population is Spanish video game players and users of video game live streaming services between the ages of 14 and 24. According to the Spanish Association of Video Games, in 2018, around 3.4 million users in Spain were in this age group [65]. A convenience sampling approach was employed in an effort to obtain a broad sample. We contacted several educational and university centres, providing a link to the questionnaire, the introduction to which stated that participation was voluntary, and that data processing was authorised exclusively for research purposes. The questionnaire was also distributed through social networks, video game forums, and Spanish streamers’ Twitch channels. The study was carried out in line with the ethical recommendations proposed in previous research in the same field [64,66], and observing the principles and recommendations of the Declaration of Helsinki [67].

### 2.2. Measurement Instrument

The questionnaire design was based on previous research papers [43,44,47,68], adapted in order to gain an understanding of the determinants of the use of these platforms, and assess the benefits and disadvantages.

The questionnaire was divided into five blocks:

a. Sociodemographic characteristics.
b. Gamer attributes, preferred platforms for gaming/viewing, time spent weekly, and self-perception of skill level.
c. Motivations for gaming/viewing content.
d. Potential harm.
e. Potential benefits.

The sociodemographic section included closed-ended questions, with a number of answer choices for the respondents to choose from. Subsequent blocks included questions assessed using a 5-point Likert scale. Before the final survey, a pre-test was performed on a control group (*n* = 12), in order to correct any phrasing or inconsistencies, as necessary. To obtain a diversity of usage habits, the selection criteria for the survey respondents were not restrictive, that is, all participants who reported having used live streaming services in the previous 6 months were included, giving rise to a high degree of randomness. The survey remained open from September to November 2019. A total of 609 questionnaires were completed, of which 580 were valid. A number of those respondents left some questions unanswered, which is why some of the tables presented do not add up to 580 users.

The sociodemographic variables include age, gender, educational level, and employment status. The set of variables focused on player attributes were selected according to the research objectives and include weekly hours spent gaming/viewing, self-perception of skill level, and preferred platforms for gaming (PC, Smartphone, Tablet, PlayStation, Xbox, Nintendo).

Regarding the motivation variables, drawing on the classification proposed by Sjoblom and Hamari [43], they were categorised into four groups: information-seeking motivation, with three items (to follow tournaments or events, to learn gaming strategies, to stay up to date on video games), entertainment motivation, two items (for entertainment, as a complement/alternative to social networks), social motivation, with three items (to communicate with others in the chat room, to watch Twitch/platforms with friends, to contact streamers), and social-integrative motivation, with two items (to be part of the gaming community, to be part of today’s gaming culture). In this section, the scales used were those employed in the questionnaires of Gros, Wanner, Hackenholt, Zawadzki, and Knautz [44] and Gandolfi [47].

The potential benefits of live streaming platforms have not yet been established and more research is needed in this regard; nevertheless, some benefits are understood to be based on the unique features of these platforms, such as interactivity [40] and advantages for learning [32]. For this reason, two items were used (to help my education, for example, in languages, and to meet new friends). At the same time, the possibility was assessed of including a third item related to the potential for professionalising their hobby [21,28,69]. However, this variable may not be entirely straightforward, as the consequences of not achieving this goal could potentially be negative; as such, it was decided not to include it in the analysis for identifying user groups but rather to study it independently.

Lastly, to determine the potential adverse effects, the scale developed by Andreassen, Billieux, Griffiths, Kuss, Demetrovics, and Mazzoni [68] was adapted, with four items included: I often neglect important tasks to play or watch others play, I spend more and more time gaming or watching others play, I have felt bad when I could not play or watch, and I play or watch others to forget about my problems. Furthermore, three specific items related to the possible aggressive use of these services [70,71] were added: I have made hurtful comments, I have received hurtful comments, and I use platforms to troll other users.

### 2.3. Statistical Analyses

Once the data had been tabulated, a reliability analysis was performed, using Cronbach’s Alpha to evaluate the validity of the responses and the measurement scale. In all cases, the value of this coefficient was greater than 0.8, indicating good internal consistency. Given the number of variables, an exploratory/confirmatory factor analysis was then carried out, using the Varimax orthogonal rotation method. Of the 19 variables initially evaluated, 4 non-significant variables were eliminated: to contact streamers, to watch Twitch/platforms with friends, to be part of the gamer community, to be part of today’s gaming culture, and the remaining ones were reduced down to four factors.

On the basis of these factors, an analysis was carried out to identify possible patterns according to which survey respondents could be grouped. User subgroups were determined using non-hierarchical clustering techniques. Thus, cluster analysis was applied to determine whether there are characteristic features that can define their behaviour. The results of the dendrogram and the icicle plot suggested an optimal solution consisting of four clusters. To check the significance of belonging to a particular cluster of individuals, as indicated by the evaluation of the variables, a MANOVA (Multivariate analysis of variance) analysis was used. In addition, since this analysis cannot be used to determine where the significant differences in means exist, Tukey’s post hoc test was applied. The statistical analyses were carried out using IBM SPSS Statistics 25 (IBM Corp. Released 2017. IBM SPSS Statistics for Windows, Version 25.0. IBM Corp, Armonk, NY, USA).

## 3. Analysis and Results

Table 1 shows the sociodemographic variables, where it can be seen that the male gender predominates, and that notably more time each week is spent playing than viewing. A relationship was detected between the variable age and gaming hours (contingency coefficient = 0.330; 0.00), as well as between age and viewing hours (contingency coefficient = 0.287; 0.00), with younger respondents registering more hours. For this reason, it was decided to divide age into two groups in the subsequent cluster analysis (19 and under and 20–24 years old). In addition, a relationship was found between gender and gaming hours (contingency coefficient = 0.468; 0.00) and gender and viewing hours (contingency coefficient = 0.370; 0.00), with male respondents registering more hours.

Exploratory factor analysis (EFA) [72] was then applied, with the aim of computing the factor scores of the identified factors in order to determine their strength in the behaviour of users of these types of platforms. The results of the analysis (Table 2) yielded four factors, characterised as follows:

- Recreational-informative factor: incorporates the motivations linked to utility, connected to the needs they meet. This factor explains 28.9% of the total variance in gaming/viewing on streaming platforms.
- Escapist-addictive factor: incorporates aspects considered potentially negative in the use of video games and social networks [56,60]. This factor accounts for 18.4% of the total variance.
- BM factor (bad manners or bad behaviour): includes potentially negative issues related to the aggressiveness arising in relationships with third parties when interacting on such platforms, such as making or receiving hurtful comments or trolling others, and it explains 12.5% of the variance.
- Social factor: incorporates issues connected to social networks within these platforms, and accounts for 12.1% of the total variance explained.

In order to evaluate the validity and reliability of the four dimensions extracted in the EFA, we used confirmatory factor analysis (CFA) with structural equation modelling, applying the unweighted least squares (ULS) estimation method. Table 3 shows the Bentler (CFI) (Compartive Fit Index), Tucker Lewis (TLI), and Root Mean Square Error of Approximation (RMSEA) indices. All the measurements are within the range to be considered a good fit [73].

**Figure 1:**
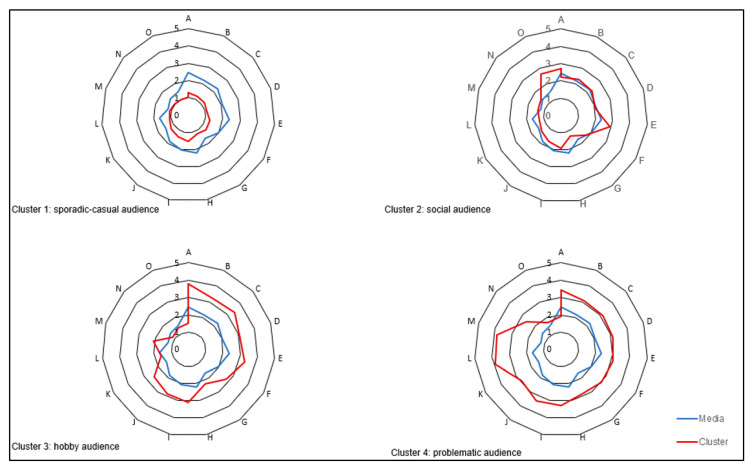
Profiles of gamer/viewer subgroups (the cluster is in red, the sample mean in blue).

The factor scores indicated the use of a non-hierarchical clustering method, with the aim of maximising the variance between groups and minimising it within each group. Of all the possible solutions, the one that best met these criteria was chosen and four clusters were established, and the user profiles in the detected groups are shown in Figure 1 (the cluster is in red, the sample mean in blue).

The tests for differences between means highlighted that belonging to a particular group has a significant relationship with the factors considered (recreational-informative, escapist-addictive, BM, and social: significant differences at the 0.05 level between the four measures, or at least in three), yielding four possible audience profiles:

- Cluster 1 denoted a sporadic-casual audience: the largest group with 256 respondents or 44.1% of the sample, and this group scored the lowest in all the dimensions analysed.
- Cluster 2, or social audience: composed of 82 respondents (14.1%), it registered high scores in social variables and moderate scores in the recreational-informative dimension.
- Cluster 3, termed a hobby audience: comprising 205 respondents (35.3%), their motivation stems from aspects such as entertainment, learning strategies, staying up to date on video games, and complementing social networks. They show medium–high values in components related to the escapist-addictive factor, such as playing to forget problems or spending an ever-increasing amount of time gaming.
- Cluster 4, or potentially problematic audience: composed of 37 users, only 6.4% of the sample; however, this cluster represents the most complete spectrum, registering very high scores in factors associated with bad behaviour in networks and notable escapist-addictive elements, together with high recreational-informative motivation.

The groups were then characterised according to factors associated with problematic gaming, such as gender, age, device, gaming/viewing time [56], as well as other variables such as self-perception of skill level as a player, or whether they identify possibilities for turning their video game hobby into a profession. A contingency table analysis and Pearson’s χ2 test (Table 3) were applied to all of them. The variable preferred type of video game was studied independently.

The results showed that young women and female adolescents feature more prominently in Clusters 1 (sporadic-casual) and 2 (social). They have a smaller presence in Cluster 3 (hobby) and only a minimal presence in Cluster 4 (problematic). The male survey respondents are mostly found in Clusters 3 and 4, more closely related to the concept of gaming as a hobby and problematic gaming. Furthermore, Clusters 3 and 4 are made up of the youngest users (under 19 years of age), in contrast to Clusters 1 and 2, which comprise respondents aged between 20 and 24 years old. Weekly hours spent gaming or viewing are also related to the allocation to the different clusters: only 2% of those in Cluster 1 admit to playing more than 15 h a week, whereas this proportion increases to 32.4% in Cluster 4. A similar pattern is observed with viewing. The young people included in Clusters 3 and 4 perceive themselves as having a higher skill level as a player than those belonging to Clusters 1 and 2. Regarding devices, it can be seen that the use of PCs and PlayStations is more widespread in Clusters 3 and 4, while no significant relationships were found for the use of tablet. On the other hand, devices such as Xbox or Nintendo have a negligible presence in the sample. Another noteworthy aspect is that the members of Clusters 3 (27%) and 4 (15.2%) think that their passion for video games can be turned into a profession. Lastly, it is worth noting that action/adventure was the favourite genre in all groups: a preference for potentially more addictive genres appeared in Clusters 3 and 4, with high percentages of MMORPG/FPS/MOBA (Massively Multiplayer Online Role-Playing Game, First Person Shooter, Multiplayer Online Battle Arena), which were not seen in Clusters 1 and 2.

Regarding the characterisation of the groups, Table 4 shows the mean values for the items considered: self-perception of skill level as a player, perceptions with respect to professionalising their video games hobby, or beliefs about the educational benefits to be gained from streaming platforms (for example, with respect to languages). As can be seen, Cluster 3 and particularly Cluster 4 stand out in all of these, which is to be expected as they are the ones who devote the most time to gaming and viewing. However, the progressive increase in the mean values across the four clusters is striking. Furthermore, the mean values registered in Clusters 3 and 4 indicate that those users who consider themselves to be very experienced (well above the average) are precisely those who think that viewing content is beneficial to their learning, and to a lesser extent, see it as feasible to dedicate themselves professionally to activities related to gaming, including video game streaming (Table 5).

To determine the extent to which the four clusters differ from one another with respect to these three variables, post hoc multiple comparisons tests were used, namely the Brown-Forsythe and Welch’s t tests (Table 6). In this case, significant differences were detected between the four clusters for the variable self-perception of skill level as a player. The other two variables analysed showed substantial and significant differences between Clusters 1 and 2, on the one hand, relative to Clusters 3 and 4 on the other; moreover, there are no significant differences between Clusters 3 and 4.

## 4. Discussion and Conclusions

The main objective behind this study was to gain an understanding of the characteristics of young people and adolescents’ entry into live video game streaming services. To that end, their motivations were determined following previous research on motivation and adverse effects related to addictive use [21,44,47,68,69]. To establish the different groups of users, variables associated with users’ behaviour on these types of services were also considered, including making or receiving hurtful comments or using the platforms to troll other users, as well as perceptions about possible benefits related to learning or the possibility of turning a video game or streaming hobby into a professional opportunity.

Four clusters were identified through factor analysis followed by clustering. The first contained the most users and registered the lowest scores in all the variables analysed. Denoted the sporadic-casual audience, it fits a profile of users who are newcomers to these platforms and whose main motivation is entertainment and spending time enjoying their favourite hobby without the need to perform [47]. The time they spend gaming/viewing as well as their self-perception of their skill level as a player indicates that these are users who would only use such services occasionally. The second cluster, denoted the social audience, is notable for containing a larger number of female users. The time they spend gaming/viewing is slightly higher than the previous cluster, and they registered higher social valuations than all the other clusters. This characteristic is in line with the findings reported by Hilvert-Bruce, Neill, Sjoblom, and Hamari [49], and is a reflection of the importance for certain individuals of interaction with others as a primary motivation for using this type of service. The average age of the respondents in the first two clusters was considerably higher than that of the others, and this could be an indication that as users’ age increases, their motivation for using these platforms changes, with social motivations corresponding to higher age brackets. For example, Long and Tefertiller [74] found correlations between real-life communication, partnership seeking, and social interactions in a sample with an average age above 30 years old. However, possible cohort effects [75,76] should be considered, as younger users grow up in a digital world where video game streaming is more commonplace, while older users had a different experience growing up, which could be reflected in this result. The third cluster, which is the second largest, was made up of younger individuals, motivated mainly by meeting the need for information about their favourite hobby. Their self-perception of their level as a player is high, and social motivations take a back seat, probably due in part to the fact that these users turn to streaming services to improve their abilities as a player [45], that is, interaction would be supplemental to the acquisition of new skills acquired watching others play. The last of the clusters, the smallest, revealed a worrying spectrum: it contained the users who spend the most time playing and watching others play, they are mostly under 19 years old, consider themselves expert or professional players, use the platforms mainly to be informed, and are notable for registering high values associated with bad behaviour in the networks [77].

Focusing attention on the motivation variables used in the different clusters, the uses and gratifications theory has often been used to gain a better understanding of the emerging phenomenon of video game live streaming [17,37,43,44,45,48,49,78,79,80]. The profiles of the four groups presented align with the findings reported by Sjoblom and Hamari [43], in that the most intensive users are those who attach greater importance to the search for information [45]; this type of motivation, along with a lack of self-control and self-esteem, is found to be a notable predictor of problematic use of live streaming services [64]. Indeed, Cluster 3 and particularly Cluster 4 are the ones that present the highest values for the information-seeking motivation. However, Chen and Chang [18] analysed the moderating effects of the information-seeking motivation and escapism on problematic use, finding no significant relationship in the profiles displaying high levels of this type of motivation. All this highlights the enormous complexity of the uncontrolled use of these services and the number of variables that could influence it, thus underscoring the need for more empirical evidence.

Regarding the variables capturing users’ perceptions, their self-perception of their skill level as a player was particularly notable for the scores registered by the four clusters, with the younger players grouped in Clusters 3 and 4 recording the highest levels. According to White [81] and Hopp et al. [82], motivations are driven by competitiveness and progress towards the acquisition of skills and knowledge; indeed, it is users in the latter two clusters that reported the highest values for how platforms improve their learning skills, and are also the ones that see the greatest possibilities of turning their hobby into a profession. This finding is significant given that Cluster 4—considered potentially problematic—is particularly notable in this regard, which could imply that it is the levels of competitiveness driving their bad behaviour in the networks.

### Limitations and Future Directions

The classification established here is a reflection of some of the existing groupings for types of players [46,83,84,85]. The results highlight the need to consider the player profile in motivational analyses of live streaming platforms, since users do not stop being players on becoming a viewer [43]: it seems that their player profile marks their behaviour as a viewer. However, this study is not without limitations. First, the methodology used involved a convenience sample of Spanish young people and teenagers who accessed the questionnaire. Despite the degree of randomness in the sample and the fact that the profile of the sample coincides with that reported by AEVI [65] (The Spanish Video Game Association), the exact size of the reference population is not known. Applied to the Spanish population between those ages [86], we could consider the sample universe to be 3.4 million players; thus, the margin of error for a random sample could be around 4.07% with a confidence level of 95%. Second, this study is conceived of as an initial approach to the phenomenon, it reveals a static snapshot of the emergence of these types of services. It is precisely this limitation that opens up new avenues for a broader longitudinal analysis of specific user groups. Third, the use of variables such as users’ self-perception of their skill level as a player and their behaviour, as well as the relationships that can be identified with other variables, merit further investigation in the future. In the present study, these variables were based on self-reported data, which entails the standard limitations regarding respondents having overly positive perceptions of themselves or differences in their assessments of negative effects. Furthermore, the questions used in the measurement instrument rely on scales used in previous studies [43,44,47,68]; thus, the application of these scales to the field of video game live streaming needs to be validated.

## 5. Conclusions

Unlike previous studies such as those by Sjoblom and Hamari [43] and Wang, Tian, Lan, Yang, and Zhang [14], the present study identified user profiles based on their motivation, perceptions about problematic use, and potential benefits. The results of our research show that as the intensity of use increases, for both video games and streaming platforms, there is an increase in possible problematic behaviour and self-reported perceptions about the perceived benefits. Female and older users feature more prominently in the casual and social groups. Conversely, in the groups that spend more time playing/watching, we see more male and younger users (hobby/problematic). These results are in line with those reported by Andreassen et al. [87] in their analysis of the relationships between the addictive use of social networks and video games. They thus represent an initial attempt to examine the potential positive and negative effects, thereby contributing to the limited research to date focused on the problems arising from the use of this new type of platform [18,64].

Lastly, we believe that this research can help parents, caregivers, and guardians identify certain profiles, offering a better understanding of a phenomenon that is here to stay, and helping to strike the difficult balance between advantages and disadvantages. Generally speaking, except for the problematic profile (a minority), the motivations relating to entertainment and learning new strategies or gaming techniques are those that register the highest scores.

## Figures and Tables

**Table 1:** Sociodemographic profile.

Variable	*n*	%
**Age**		
16 and under	94	16.2
17–19 years old	153	26.4
20–22 years old	219	37.8
23–24 years old	114	19.7
**Weekly gaming hours**		
0–3 h	288	49.7
3–7 h	104	18.0
7–10 h	68	11.7
10–15 h	46	7.9
15–25 h	42	7.3
More than 25 h	31	5.4
**Weekly viewing hours**		
0–3 h	398	69.1
3–7 h	83	14.4
7–10 h	43	7.5
10–15 h	26	4.5
15–25 h	13	2.3
More than 25 h	13	2.3
**Gender**		
Female	237	40.9
Male	343	59.1
**Educational level**		
Primary education	67	11.6
Secondary education	192	33.1
Upper Secondary/VET	257	44.3
University degree	56	9.6
Postgraduate/Doctorate	8	1.4
**Employment status**		
Full-time work	20	3.5
Part-time work	10	1.7
Work and study	91	15.7
Study	451	78.0
Unemployed	6	1.0

VET, Vocational education and training.

**Table 2:** Rotated component matrix. Gaming/viewing motivation variables.

Variables	Components				Factors
	1	2	3	4	
For entertainment—A	0.83				Recreational-informative
To learn gaming strategies—B	0.81				
To stay up to date on video games—C	0.81				
To follow tournaments or events—D	0.78				
As a complement/alternative to social networks/Tv—E	0.76				
To help my education (languages)—F	0.66				
I have felt bad when I could not play—G		0.81			Escapist/addictive
I play to forget about my problems—H		0.74			
I spend more and more time gaming—I		0.69			
I often neglect important tasks to play—J		0.68			
I have made hurtful comments—K			0.84		BM *
I have received hurtful comments—L			0.70		
I use platforms to troll other users—M			0.65		
I use platforms to meet new friends—N				0.89	Social
I use platforms to communicate with others in the chat room—O				0.83	
Eigenvalues	4.33	2.75	1.88	1.81	
% Variance	28.89	18.39	12.55	12.09	
Cumulative % variance	28.89	47.28	59.84	71.94	
Sampling adequacy Kaiser–Meyer–Olkin (KMO): 0.898					
Bartlett’s Test of Sphericity χ^2^ = 5145.127;d.f: 105; *p* 0.000					

* BM = Bad manners, d.f. = d.f = degrees of freedom, *p* = value.

**Table 3:** Fit indices, confirmatory factor analysis (CFA).

Model	Incremental		Global		
	CFI	TLI	GFI	RMSEA (IC 90%)	SRMR
Total	0.996	0.995	0.996	0.047 (0.038–0.056)	0.046
Sample 1	0.994	0.992	0.993	0.058 (0.045–0.071)	0.055
Sample 2	0.999	0.999	0.994	0.020 (0.000–0.039)	0.053

**Table 4:** Characterisation of components.

Variable	Categories	C1	C2	C3	C4	*n* *	χ^2^	Sig.
		*n* = 256 (44.1%)	*n* = 82 (14.1%)	*n* = 205 (35.3%)	*n* = 37 (6.4%)			
Gender	Female	158 (61.7%)	38 (46.3%)	38 (18.5%)	3 (8.3%)	237	105.812	<0.001
	Male	98 (38.3%)	44 (53.7%)	167 (81.5%)	34 (91.7%)	343		
Age	19 and under	77 (30.1%)	26 (31.7%)	122 (59.5%)	22 (59.5%)	247	48.679	<0.001
	20–24 years old	179 (69.9%)	56 (68.3%)	83 (40.5%)	15 (40.5%)	333		
Gaming hours	0–7 h	230 (90.2%)	67 (81.7%)	84 (41.0%)	11 (29.7%)	392	162.076	<0.001
	7–15 h	20 (13.4%)	11 (13.4%)	69 (33.7%)	14 (37.8%)	114		
	More than 15 h	5 (2.0%)	4 (4.9%)	52 (25.4%)	12 (32.4%)	73		
Viewing hours	0–7 h	243 (96.0%)	75 (91.5%)	140 (68.3%)	23 (62.2%)	481	82.089	<0.001
	7–15 h	8 (3.2%)	5 (6.2%)	47 (22.9%)	9 (24.3%)	69		
	More than 15 h	2 (0.8%)	1 (1.2%)	18 (8.8%)	5 (13.5%)	26		
Player skill level	Novice/amateur	170 (66.4%)	37 (45.7%)	30 (14.8%)	1 (2.7%)	238	187.655	<0.001
	Regular	63 (25.1)	30 (37.0%)	83 (40.9%)	11 (29.7%)	187		
	Expert/Pro	18 (7.0%)	14 (17.1%)	90 (44.3%)	25 (67.6%)	147		
PC	None–A little	216 (84.7%)	59 (72.5%)	99 (48.3%)	16 (43.2%)	390	79.830	<0.001
	Quite a lot/A lot	39(15.3%)	22 (27.2%)	106 (51.7%)	21 (56.8%)	188		
Smartphone	None–A little	153 (59.8%)	33 (40.2%)	79 (38.7%)	14 (38.9%)	279	24.375	<0.001
	Quite a lot/A lot	103 (40.2%)	49 (59.8%)	125 (61.3%)	22 (61.1%)	299		
PlayStation	None–A little	226 (89.0%)	67 (82.7%)	121 (59.3%)	18 (50.0%)	432	68.037	<0.001
	Quite a lot/A lot	28 (11.0%)	14 (17.3%)	83 (40.7%)	18 (50.0%)	143		
Tablet	None–A little	237 (92.9%)	74 (91.4%)	185 (90.7%)	31 (86.1%)	527	2.199	<0.532
	Quite a lot/A lot	18 (7.1%)	7 (8.6%)	19 (9.3%)	5 (13.9%)	49		
Xbox	None–A little	248 (96.9%)	75 (93.8%)	194 (95.0%)	31 (86.1%)	548	12.108	<0.007
	Quite a lot/A lot	5 (2.0%)	5 (6.3%)	10 (4.9%)	5 (13.9%)	25		
Nintendo	None–A little	243 (95.3%)	76 (93.8%)	175 (85.8%)	28 (77.8%)	522	20.141	<0.001
	Quite a lot/A lot	12 (4.7%)	5 (6.2%)	29 (14.2%)	8 (22.2%)	54		
Hobby/profession	None/Hardly any	254 (99.2%)	70 (86.4%)	140 (68.6%)	24 (64.9%)	488	101.622	<0.001
	Some	2 (0.8%)	3 (3.7%)	33 (16.2%)	3 (8.1%)	41		
	Quite a lot/A lot	0 (0%)	8 (9.9%)	31 (15.2%)	10 (27.0%)	49		

* There are groups that do not add up to 580 users because some respondents did not provide answers to all the options. Sig. = significance.

**Table 5:** Characterisation of the three perception variables.

Variable	Cluster				ANOVA	
	C1	C2	C3	C4	F	Sig.
	*n* = 256	*n* = 82	*n* = 205	*n* = 37		
Streaming is beneficial to my education	1.31 ^(^**^)^	1.84 ^(^**^)^	2.75 ^(^*^)^	3.00 ^(^*^)^	100.603	<0.001
Professionalise my hobby	1.07 ^(^**^)^	1.62 ^(^**^)^	2.08 ^(^*^)^	2.32 ^(^*^)^	55.024	<0.001
Self-perception of skill level as a player	1.86 ^(^**^)^	2.38 ^(^**^)^	3.35 ^(^**^)^	3.95 ^(^**^)^	93.988	<0.001

(**) Significant difference at 0.05 between the four cluster means; (*) significant difference at 0.05 between at least three cluster means. ANOVA = analysis of variance.

**Table 6:** Robust tests of homogeneity of variance and equality of means for perception variables.

Variables	Homogeneity of Variances (Levene)		Equality of Means		
Streaming is beneficial to my education	23.39	<0.001	Welch	100.848	<0.001
			Brown-Forsythe	76.716	<0.001
Professionalise my hobby	112.016	<0.001	Welch	59.442	<0.001
			Brown-Forsythe	36.598	<0.001
Self-perception of skill level as a player	5.802	<0.005	Welch	99.963	<0.001
			Brown-Forsythe	95.709	<0.001

## Data Availability

The data used to support the findings of this study are available from the corresponding author upon request.
